# The development of sensorimotor cortical oscillations is mediated by pubertal testosterone

**DOI:** 10.1016/j.neuroimage.2022.119745

**Published:** 2022-11-09

**Authors:** Madison H. Fung, Elizabeth Heinrichs-Graham, Brittany K. Taylor, Michaela R. Frenzel, Jacob A. Eastman, Yu-Ping Wang, Vince D. Calhoun, Julia M. Stephen, Tony W. Wilson

**Affiliations:** a Institute for Human Neuroscience, Boys Town National Research Hospital, Boys Town, NE, USA; b Department of Pharmacology & Neuroscience, Creighton University, Omaha, NE, USA; c Department of Biomedical Engineering, Tulane University, New Orleans, LA, USA; d Tri-institutional Center for Translational Research in Neuroimaging and Data Science (TReNDS), Georgia State University, Georgia Institute of Technology, Emory University, Atlanta, GA, USA; e Mind Research Network, Albuquerque, NM, USA

**Keywords:** Beta, Puberty, Magnetoencephalography, Motor, Hormones

## Abstract

Puberty is a period of substantial hormonal fluctuations, and pubertal hormones can modulate structural and functional changes in the developing brain. Many previous studies have characterized the neural oscillatory responses serving movement, which include a beta event-related desynchronization (ERD) preceding movement onset, gamma and theta responses coinciding with movement execution, and a post-movement beta-rebound (PMBR) response following movement offset. While a few studies have investigated the developmental trajectories of these neural oscillations serving motor control, the impact of pubertal hormone levels on the maturation of these dynamics has not yet been examined. Since the timing and tempo of puberty varies greatly between individuals, pubertal hormones may uniquely impact the maturation of motor cortical oscillations distinct from other developmental metrics, such as age. In the current study we quantified these oscillations using magnetoencephalography (MEG) and utilized chronological age and measures of endogenous testosterone as indices of development during the transition from childhood to adolescence in 69 youths. Mediation analyses revealed complex maturation patterns for the beta ERD, in which testosterone predicted both spontaneous baseline and ERD power through direct and indirect effects. Age, but not pubertal hormones, predicted motor-related theta, and no relationships between oscillatory responses and developmental metrics were found for gamma or PMBR responses. These findings provide novel insight into how pubertal hormones affect motor-related oscillations, and highlight the continued development of motor cortical dynamics throughout the pubertal period.

## 1. Introduction

The pubertal period of development consists of many physiological changes, including increased production of the sex steroids testosterone and estradiol, which facilitate sex-specific developmental processes (Sisk and Foster, 2004). These hormones induce molecular and cellular processes such as neurogenesis, cell survival, synaptic pruning, and programmed cell death to influence cortical structural changes (Ahmed et al., 2008; Davis et al., 1996; Nuñez et al., 2002), which are thought to result in sexually dimorphic patterns of grey and white matter development (De Bellis, 2001; Giedd et al., 1999; Gogtay et al., 2004; Shaw et al., 2008). Of note, intracellular androgen receptors are present throughout the human cerebrum, but are particularly densely distributed within the motor and somatosensory cortices (Kritzer, 2004). Further, the structural growth trajectories of these posterior frontal and parietal regions have been associated with levels of endogenous pubertal hormones in both males and females (Bramen et al., 2012; Koolschijn et al., 2014; Neufang et al., 2009; Nguyen, 2018; Paus et al., 2010; Peper et al., 2009; Vijayakumar et al., 2018).

The sensorimotor cortices are also known to be critical for generating the neural oscillations that serve motor control, which have been extensively characterized in healthy adults. Briefly, an event-related desynchronization (ERD) within the beta range (14–30 Hz), beginning prior to movement onset and remaining active through the duration of the movement, has been consistently associated with movement planning and execution (Cheyne, 2013; Gaetz et al., 2010; Heinrichs-Graham et al., 2016, 2018b, 2020; Heinrichs-Graham and Wilson, 2015, 2016; Spooner et al., 2021b; Trevarrow et al., 2019; Wiesman et al., 2020; Wilson et al., 2010, 2014). Following this response, there is a resynchronization in the beta band, termed the post-movement beta rebound (PMBR), which is thought to be instrumental in functional inhibition of the motor system and/or sensory reafference (Fry et al., 2016; Gaetz et al., 2010; Heinrichs-Graham et al., 2017; Jurkiewicz et al., 2006; Wilson et al., 2011a,b). Other motor-related oscillatory responses include the movement-related gamma (>30 Hz) synchronization (MRGS) and theta (3–6 Hz) synchronizations that occur at movement onset and are thought to index motor execution and higher order processing (Cheyne et al., 2008; Heinrichs-Graham et al., 2018a; Karakaş, 2020; Muthukumaraswamy, 2010; Spooner et al., 2021c, 2021a, 2020; Wiesman et al., 2021, 2020; Wilson et al., 2010).

Major neurophysiological changes are known to occur in the motor cortices across the lifespan (Heinrichs-Graham et al., 2018b; Heinrichs-Graham and Wilson, 2016; Rossiter et al., 2014; Spooner et al., 2021a), and there is a growing wealth of knowledge documenting developmental changes in the neural oscillatory dynamics serving motor control in youth (Gaetz et al., 2010; Heinrichs-Graham et al., 2020, 2018b; Kurz et al., 2016; Trevarrow et al., 2019; Wilson et al., 2010). Importantly, these studies have only used chronological age as a metric for development. Given the known impact of pubertal hormones on cortical structure in somato-motor areas, and the vast individual variability in the timing and tempo of pubertal maturation (Blakemore et al., 2010; Sisk and Foster, 2004), there is a critical need for studies that disentangle chronological age and pubertal effects on cortical maturation, especially within the domain of motor control.

To date, most neuroimaging studies investigating pubertal hormone changes have largely focused on the maturation of social and affective processes using fMRI (for review, see (Vijayakumar et al., 2018)). Far fewer studies have examined how hormones influence neural oscillations serving cognitive performance (Fung et al., 2020, Fung et al., 2022) and any divergence from age-specific effects on oscillatory brain development (Embury et al., 2019; Fung et al., 2021; Killanin et al., 2020; Ott et al., 2021; Taylor et al., 2021, 2020). Thus, pubertal hormones may also uniquely impact the development of motor cortical oscillations distinct from other developmental metrics like chronological age. In the present study, we use magnetoencephalography (MEG) to study the development of volitional motor responses in relation to endogenous pubertal testosterone levels in a group of typically-developing children and adolescents. We focused on testosterone levels in this study because this hormone has been tightly linked to pubertal maturational changes in the body and brain. Briefly, testosterone levels sharply and consistently increase across the pubertal transition and enact developmental effects distinct from other sex steroids, therefore, rising levels of testosterone can independently serve as a reliable metric of pubertal maturation in both males and females (Shirtcliff et al., 2009). To our knowledge, no previous study has examined the impact of pubertal hormones on the developmental trajectory of motor cortical oscillations, or probed how such effects may differ from the well-described age-related changes. All participants completed the motor task during MEG and the resulting neural responses were transformed into the time-frequency domain and imaged using a beamforming approach to identify the cortical origin of significant oscillatory motor responses. Virtual sensor time series were then extracted from the peak voxel of each response, and these were subjected to mediation analyses to identify the relationships between developmental metrics (i.e., testosterone levels and age) and oscillatory motor responses. Based on the existing literature demonstrating unique effects of hormones on structural brain maturation and age-related changes in oscillatory motor development, we hypothesized that testosterone levels would be coupled to neural oscillatory responses in the motor cortex, above-and-beyond the effects of chronological age alone.

## 2. Materials and methods

### 2.1. Participants

Seventy-four typically developing children and adolescents ages 9–15 years (*M*_*age*_ = 12.99 years, *SD* = 1.72; 41 females; 70 right-handed) completed a basic sensory detection task and provided saliva samples as part of the National Science Foundation-funded Developmental Chronnecto-Genomics (Dev-CoG) study (Stephen et al., 2021). All participants were recruited from the Omaha site. Of note, the raw MEG data from this sample of participants has been previously published (Fung et. al., 2021), though the present manuscript utilized a different index of development (i.e., hormones) and a complete reanalysis of all MEG data to probe a different functional construct (i.e., motor performance vs. visual processing in the previous paper). Exclusionary criteria, determined by parent report, included attention-deficit/hyperactivity disorder (ADHD) or other psychiatric or neurological disorders affecting brain function, history of head trauma, and general MEG/MRI exclusionary criteria such as the presence of metal implants, dental braces or permanent retainers, or other metallic or otherwise magnetic non-removable devices. All procedures were approved by the Institutional Review Board, and informed consent from the child’s parent or legal guardian, as well as assent from the child, were obtained before proceeding with the study.

### 2.2. Procedure

#### 2.2.1. Salivary testosterone collection and measurement

At least 2.0 ml of whole unstimulated saliva was collected from each participant. Specifically, children were asked to passively drool into an Oragene DISCOVER (OGR-500; www.dnagenotek.com) collection tube until liquid saliva (not bubble) exceeded the fill line indicated on the tube. A single-channel pipette was then used to extract 0.5 ml from the collection tube (prior to the release of the protease inhibitors for long-term storage), and this 0.5 ml was immediately transferred into a labeled micro-centrifuge tube and placed in a −20°C freezer for storage. Participants were instructed to refrain from consuming any food, liquids, or chewing gum for at least an hour before providing the saliva sample, and samples were generally collected in the afternoon to minimize circadian influences. All samples were assayed in duplicate at the University of Nebraska Lincoln Salivary Biosciences Laboratory using a commercially-available assay kit for salivary testosterone (Salimetrics; www.salimetrics.com). The testosterone assay kit had a sensitivity of 1pg/mL, with a range of 6.1–600 pg/mL. The intra- and inter-assay coefficients of variation were 5.28% and 8.93%, respectively. The average of the duplicate tests (per hormone) were used for further analyses in the present study.

#### 2.2.2. Task paradigm

**Fig. 1.**
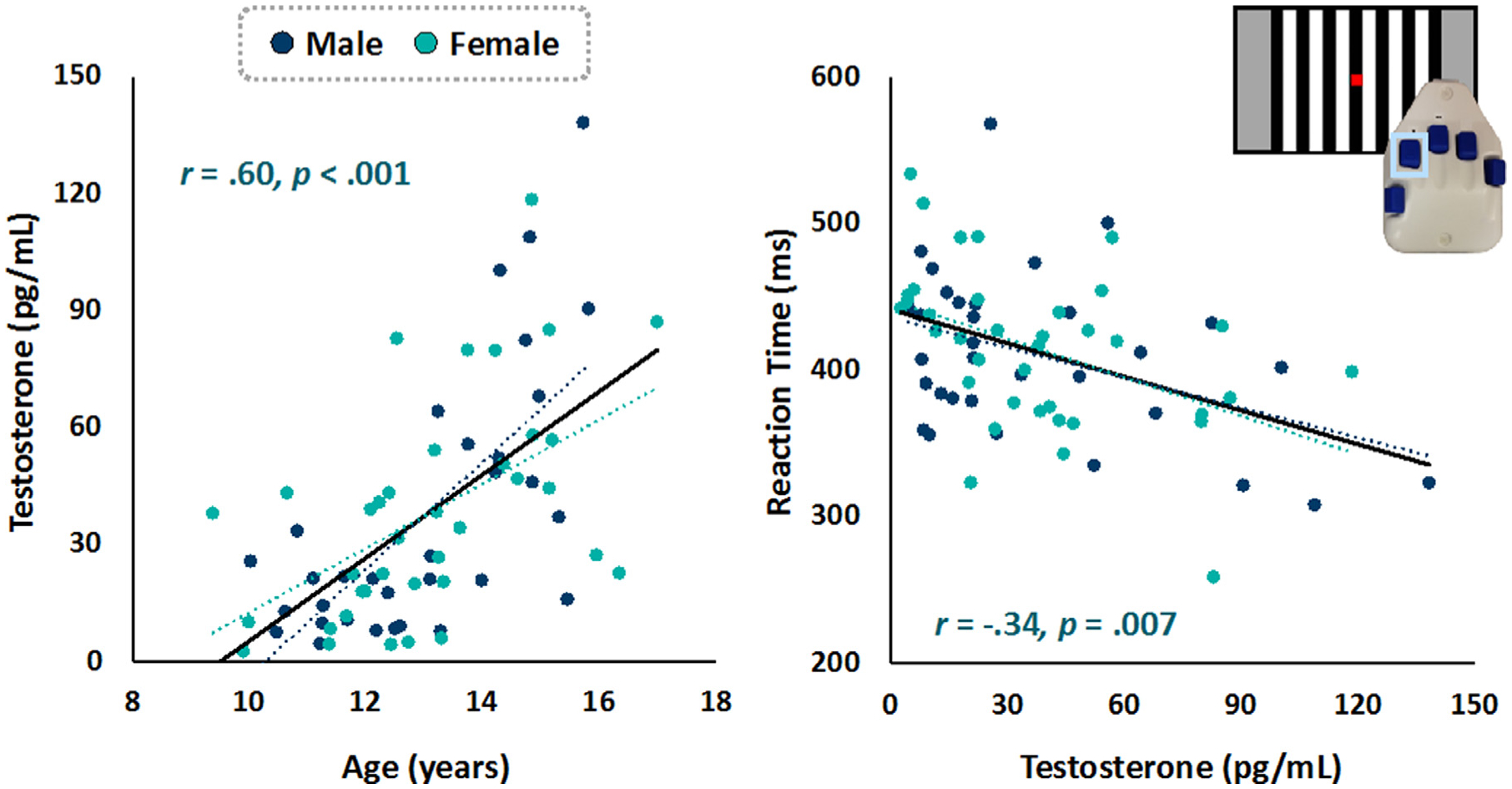
Hormone and Behavioral Results. (Left) Scatterplot shows chronological age was positively correlated with testosterone, such that older youth had higher levels of endogenous testosterone. (Right) The sensory detection task paradigm consisted of a visually-presented grating stimulus. Participants were instructed to push a button when the stimulus was detected. The scatterplot shows that more mature children with higher testosterone levels performed better on the task (i.e., had faster reaction times). This effect remained significant when controlling for age (r = −.31, p = .01). Note that males and females are plotted separately, though we did not detect significant sex differences in either correlation.

To elicit volitional motor responses, a simple sensory detection task was performed during the MEG recording (Fig. 1; see (Fung et al., 2021; Trevarrow et al., 2019)). During this task, participants were told to fixate on a centrally presented crosshair. After a fixation period of 2400–2600 ms, one of three stimuli was presented for 800 ms: a stationary visual grating stimulus, an auditory steady-state stimulus, or both. All stimuli were supra-threshold and easily detected. Participants were asked to respond with a button press using their right index finger when any stimulus was detected. Each participant performed 300 trials (100 of each condition) in a pseudo-randomized order. The total MEG task duration was 16.5 minutes. Responses with reaction times that were 2.5 SDs above or below the participant’s mean were excluded from the final analyses. Of note, given our goals and hypotheses, the present study focused on the 100 visual-only trials to minimize the spatial overlap between primary sensory and motor responses, maximize the homogeneity of the neural responses, and ensure that the longer duration auditory steady-state responses (Wilson et al., 2012, 2008, 2007) did not bias the later motor-related oscillations.

#### 2.2.3. MEG data acquisition

MEG recordings were conducted in a one-layer magnetically shielded room with active shielding engaged. Neuromagnetic responses were acquired with a MEGIN MEG system with 306 magnetic sensors (204 planar gradiometers, 102 magnetometers; MEGIN, Helsinki, Finland) using a bandwidth of 0.1–330 Hz, sampled continuously at 1 kHz. Each participant’s data were individually corrected for head motion, and noise reduction was applied using the signal space separation method with a temporal extension (tSSS; (Taulu et al., 2005; Taulu and Simola, 2006)).

#### 2.2.4. MEG Coregistration and Structural MRI processing

Preceding MEG measurement, four coils were attached to the participant’s head and localized, together with the three fiducial points and scalp surface, using a 3-D digitizer (Fastrak 3SF0002, Polhemus Navigator Sciences, Colchester, VT, USA). Once the participant was positioned for MEG recording, an electric current with a unique frequency label (e.g., 322 Hz) was fed to each of the coils. This induced a measurable magnetic field and allowed each coil to be localized in reference to the MEG sensors throughout the recording session. Since coil locations were also known in head coordinates, all MEG measurements could be transformed into a common coordinate system. With this coordinate system, each participant’s MEG data were coregistered with their individual structural T1-weighted MRI data prior to source space analyses using BESA MRI (Version 2.0). Structural T1-weighted MRI images were acquired using a Siemens Skyra 3T MRI scanner with a 32-channel head coil and a MP-RAGE sequence with the following parameters: TR = 2400 ms; TE = 1.94 ms; flip angle = 8°; FOV = 256 mm; slice thickness = 1 mm (no gap); voxel size = 1 × 1 × 1 mm. MRI, MEG, and assessment data were captured via the collaborative informatic and neuroimaging suite (COINS; (Scott et al., 2011)). These data were aligned parallel to the anterior and posterior commissures and transformed into standardized space. Following source reconstruction (i.e., beamforming), each participant’s 4.0 × 4.0 × 4.0 mm functional images were also transformed into standardized space using the transform that was previously applied to the structural MRI volume and spatially resampled.

#### 2.2.5. MEG time-frequency transformation and statistics

Cardiac and ocular artifacts were removed from the data using signal-space projection (SSP), which was accounted for during source reconstruction (Uusitalo and Ilmoniemi, 1997). The continuous magnetic time series was divided into epochs of 2200 ms duration, with a baseline extending from −1200 to −800 ms before movement onset, which was defined as 0 ms. Epochs containing artifacts (e.g., eye blinks, muscle artifacts, eye saccades, swallowing, coughing) were rejected based on a fixed-threshold method, supplemented with visual inspection. Briefly, the distribution of amplitude and gradient values per participant were computed using all trials, and the highest amplitude/gradient trials relative to the total distribution were excluded by selecting a threshold that rejected extreme values. Notably, thresholds were set independently for each participant due to differences among individuals in head size and sensor proximity, which strongly affect MEG signal amplitude. An average amplitude threshold of 1329 (SD = 307) and an average gradient threshold of 510 (SD = 204) in the sample were identified. After artifact rejection, an average of 85.5 (SD = 4.7) trials per participant were used for further analysis, and this number was not significantly correlated with chronological age (*r* = .01, *p* = .94) or testosterone (*r* = .06, *p* = .64).

Artifact-free epochs were transformed into the time-frequency domain using complex demodulation (Kovach and Gander, 2016; Papp and Ktonas, 1977), and the resulting spectral power estimations per sensor were averaged over trials to generate time-frequency plots of mean spectral density. These sensor-level data were normalized using the respective bin’s baseline power, which was calculated as the mean power during the −1200 to −800 ms time period. The specific time-frequency windows used for imaging were determined by statistical analysis of the sensor-level spectrograms across the entire array of gradiometers. To reduce the risk of false-positive results while maintaining reasonable sensitivity, a two-stage procedure was followed to control for Type 1 error. In the first stage, two-tailed paired-sample *t*-tests against baseline were conducted on each data point, and the output spectrograms of *t*-values were thresholded at *p* < .05 to define time-frequency bins containing potentially significant oscillatory deviations across all participants. In stage two, the time-frequency bins that survived the threshold were clustered with temporally and/or spectrally neighboring bins that were also below the *p* < .05 threshold, and a cluster value was derived by summing the *t*-values of all data points in the cluster. Nonparametric permutation testing was then used to derive a distribution of cluster values and the significance level of the observed clusters (from stage one) was tested directly using this distribution (Ernst, 2004; Maris and Oostenveld, 2007). For each comparison, 1,000 permutations were computed to build a distribution of cluster values. Based on these analyses, the time-frequency windows that contained significant oscillatory events across all participants were subjected to a beamforming analysis (see Results).

#### 2.2.6. MEG source imaging, virtual sensor analyses, and statistics

Cortical responses were imaged through an extension of the linearly constrained minimum variance vector beamformer (Gross et al., 2001; Hillebrand et al., 2005; Van Veen et al., 1997), which employs spatial filters in the time-frequency domain to calculate source power for the entire brain volume. The single images were derived from the cross-spectral densities of all combinations of MEG gradiometers averaged over the time-frequency range of interest, and the solution of the forward problem for each location on a grid specified by input voxel space. Following convention, we computed noise-normalized, source power per voxel in each participant using active (i.e., task) and passive (i.e., baseline) periods of equal duration and bandwidth (Hillebrand et al., 2005). Such images are typically referred to as pseudo-t maps, with units (i.e., pseudo-t) that reflect noise-normalized power differences (i.e., active vs. passive) per voxel. MEG preprocessing and imaging used the BESA (V 6.1) software.

Normalized differential source power was computed for the statistically-selected time-frequency bands (see below) over the entire brain volume per participant at 4.0 × 4.0 × 4.0 mm resolution. The resulting 3D maps of brain activity were then averaged across participants to assess the neuroanatomical basis of the significant oscillatory responses identified through the sensor-level analyses. For each significant response, virtual sensor data were extracted from the peak voxel, which corresponded to left primary motor cortex for all time-frequency windows. To create the virtual sensors, we applied the sensor weighting matrix derived through the forward computation to the preprocessed signal vector, which yielded two time series for each coordinate in source space, and then computed the vector sum of the two orientations ultimately yielding one time series per voxel per participant (Cheyne et al., 2006). The relative power (i.e., baseline-corrected) envelope was computed for the frequency bin corresponding to the derived peak using the virtual sensor time series, as well as the absolute power (i.e., not baseline-corrected) for the corresponding baseline interval to estimate the power of spontaneous activity. Amplitude values with the time window used to compute beamformer images were then averaged per oscillatory response. These response metrics were then subjected to mediation analyses to determine whether these MEG outcome variables were related to the developmental metrics. Specifically, we explored the degree to which pubertal testosterone levels mediate the effects of chronological age on spontaneous and relative motor oscillatory dynamics, which are well-established in the literature. For a more comprehensive perspective, we included the multiple mediation of age → testosterone → baseline power → relative power for all oscillatory responses, which is likely more representative of the complete active neural system (in comparison to examining either the baseline or relative power effects in isolation of one another). We utilized bias-corrected confidence intervals based on 1,000 bootstrapped samples to more robustly detect any potential relationships between brain activity and development (Austin and Tu, 2004; Efron and Tibshirani, 1986; Fritz and MacKinnon, 2007). Mediation analyses were conducted in Mplus (version 8.1).

## 3. Results

### 3.1. Demographic and hormone results

Of the 74 participants who completed the task, five were excluded due to excessively noisy MEG data or other errors in MEG data acquisition. Thus, the final sample consisted of 69 children and adolescents (*M*_*age*_ = 13.05 years, *SD* = 1.74; 37 females; 65 right-handed).

As expected, chronological age was positively correlated with testosterone levels in the whole sample (*r* = .60, *p* < .001; Fig. 1), such that older children tended to have higher levels of testosterone. We did not detect any significant sex difference in the correlation between age and testosterone levels.

### 3.2. Behavioral results

Participants performed well on the sensory detection task, responding to 97.03 ± 3.97% of trials with an average reaction time of 395.25 ± 55.10 ms. There was a significant negative correlation between age and reaction time (*r* = −.33, *p* = .006), such that older participants had faster reaction times. A negative association between testosterone and reaction time (*r* = −.34, *p* = .007; Fig. 1) was also observed and remained significant when controlling for chronological age (r = −.31, p = .01). There were no sex differences in performance, and the relationship between testosterone and reaction time did not statistically differ between males and females.

### 3.3. Neural oscillatory responses to the task

**Fig. 2.**
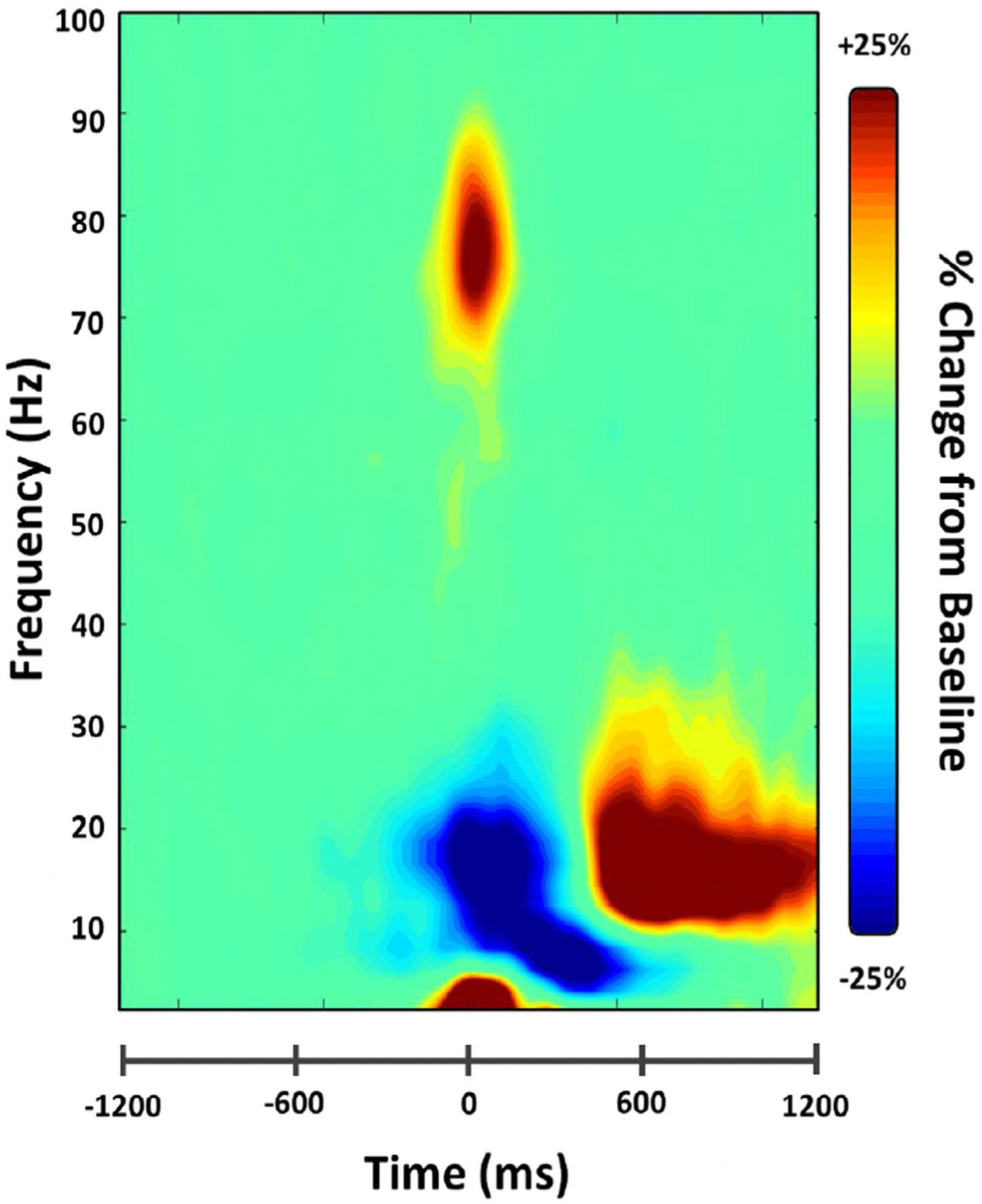
Time-frequency spectogram of motor responses. Data are displayed from a representative sensor near the left sensorimotor cortices and have been averaged across all participants. Warm colors reflect power increases relative to the baseline (i.e., synchronizations), and cool colors represent decreases relative to baseline (desynchronizations). Zero ms denotes movement onset. Time frequency windows for source imaging (beamforming) were derived from statistical analyses of these sensor-level spectrograms, which indicated significant bins in theta, beta, and gamma activity.

Statistical analysis of the time-frequency spectrograms showed significant oscillatory responses in four distinct time-frequency windows (all *p*-values < .001 after cluster-based permutation testing) across sensors near the sensorimotor cortices (Fig. 2). These corresponded to the widely known motor-related oscillations and included a significant decrease from baseline levels (i.e., desynchronization) in the beta range (18–26 Hz; beta ERD) that started −150 ms before movement onset and persisted 250 ms afterward. A significant increase from baseline beta levels (i.e., synchronization) after the movement was completed from 600–900 ms (16–22 Hz; PMBR), as well as synchronizations in the theta (3–6 Hz, −200 to 150 ms) and gamma (72–84 Hz, −50 to 75 ms; MRGS) ranges (all *p*s < .001, corrected). These four significant oscillatory responses were imaged using a beamformer, and the resulting maps were grand-averaged per oscillatory response. All four responses were tightly clustered on the left precentral gyrus, corresponding to the primary motor cortex (i.e., contralateral to a button press of the right index finger).

### 3.4. Beta ERD response

**Fig. 3.**
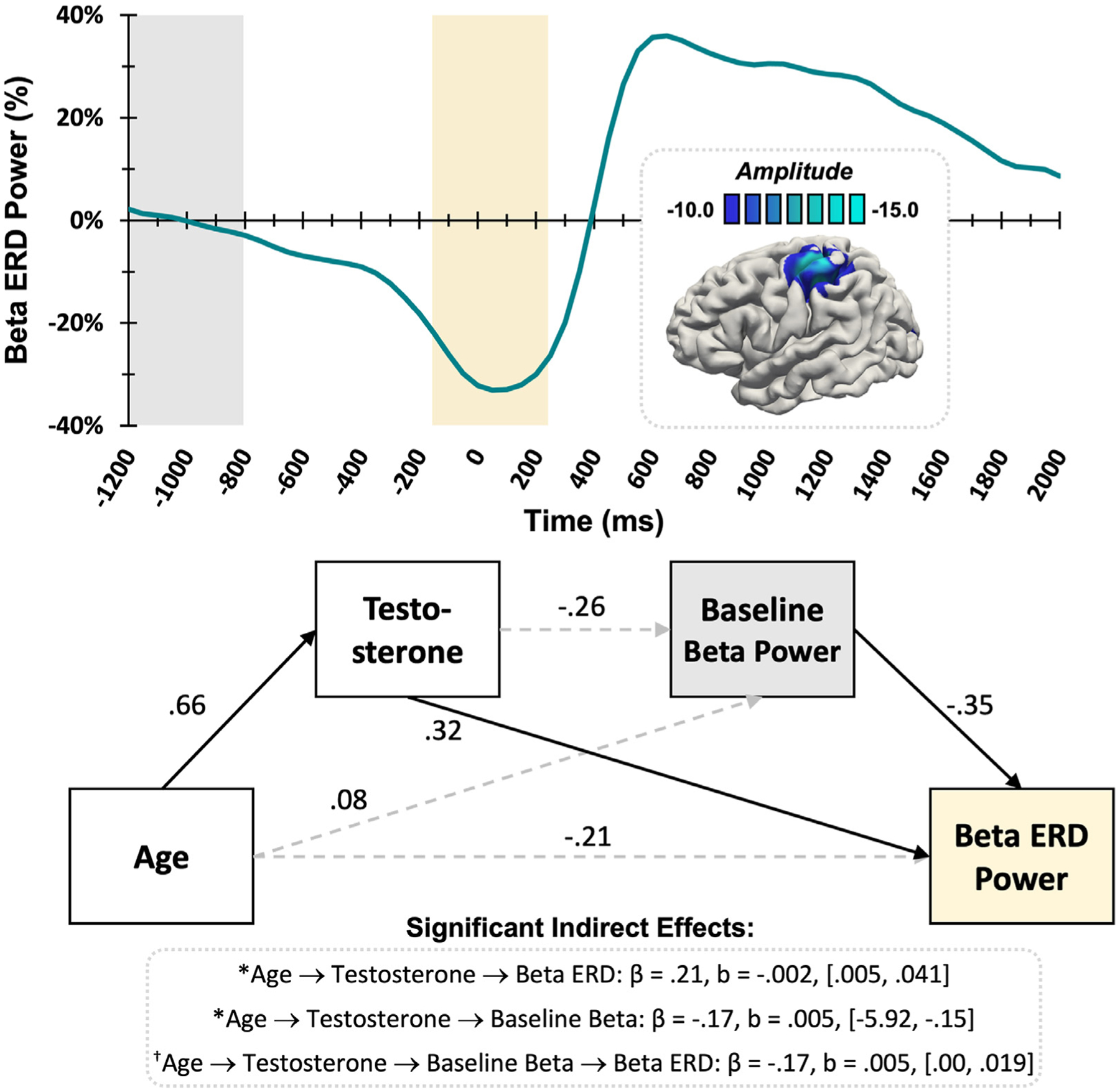
Beta ERD Response. (Top) The superimposed brain image shows that the average beta ERD response across the entire sample was centered on the contralateral primary motor cortex near the motor hand-knob feature. The color scale bar in pseudo-t units appears above the image. The time series envelope depicts changes in power relative to the baseline at the peak voxel in the beta range (18–26 Hz). The yellow box indicates the time window that was significantly different from the baseline period (grey box) based on the MEG sensor-level analyses. (Bottom) The statistical diagram demonstrates the results of the mediation analyses for the beta ERD response, with standardized coefficients shown. Direct effects are displayed graphically, where solid lines signify statistically significant relationships at p < .05 and dashed lines show nonsignificant relationships, while significant indirect effects are printed below. Testosterone significantly mediated age-related changes in the strength of both spontaneous beta activity during the baseline and beta ERD power. A full mediation was trending towards significance, where older age predicted higher testosterone, lower spontaneous beta power, and lastly weaker (i.e., less negative) beta ERD response power. * = significant, ^†^ = trending

Mediation analyses were performed to examine the relationship between development and neural oscillatory responses, which revealed that testosterone mediated the effect of age on beta ERD response metrics in the primary motor cortex (Fig. 3). Specifically, age was significantly associated with testosterone levels (*β* = .66, *p* < .001), and testosterone was related to the strength of the beta ERD response (*β* = .32, *p* = .037). Spontaneous beta power during the baseline period also predicted the strength of this subsequent beta ERD (*β* = −.35, *p* = .003), with higher spontaneous beta power predicting stronger (i.e., more negative) beta desynchronization. Further, there was a significant indirect effect of age on the strength of the beta ERD response via testosterone levels (*β* = .21, b = .002, CI [.005, .041]), such that older age predicted higher testosterone values, which ultimately predicted weaker oscillatory beta ERD responses (i.e., negative values closer to zero, or weaker desynchronization). There was also a significant indirect effect of age on spontaneous beta power via testosterone (*β* = −.17, b = −2.49, CI [−5.92, −.15]), whereby greater age predicted more testosterone, which subsequently predicted weaker spontaneous beta activity during the baseline. The direct effects of age on beta ERD power (*β* = −.21, *p* = .17) and baseline beta power (*β* = .08, *p* = .65) were not significant; thus, the model suggests that these beta oscillatory metrics were fully mediated by testosterone levels.

Of note, the full mediation with age predicting testosterone, predicting baseline spontaneous beta power, predicting beta ERD power was approaching significance (*β* = −.17, b = .005, CI [.00, .019]). The direct effect of age on beta ERD power was not significant (*β* = −.21, *p* = .17), thus the model suggests this relationship would be fully mediated by testosterone and spontaneous beta power during the baseline in a larger (better-powered) sample.

### 3.5. PMBR response

**Fig. 4.**
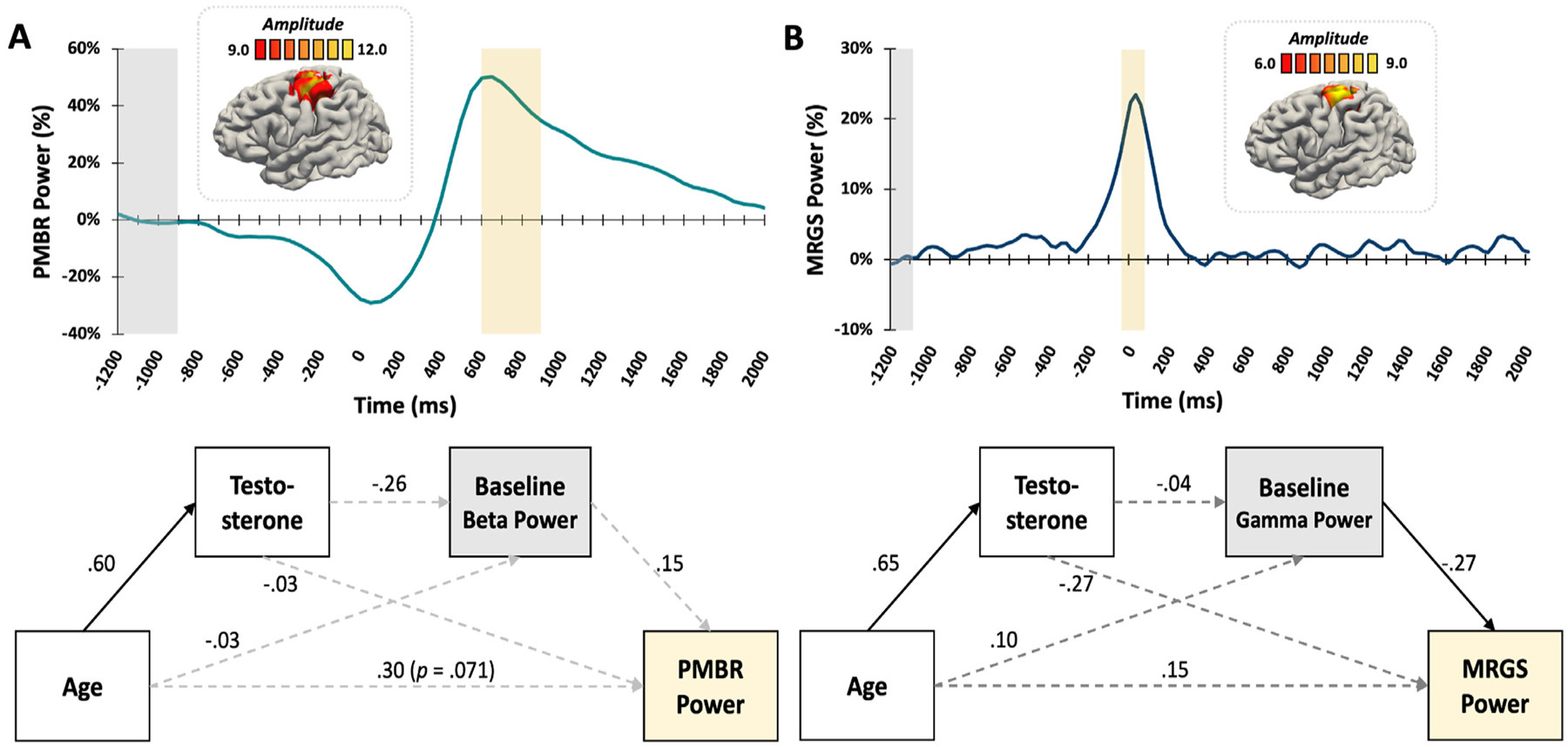
PMBR and MRGS Responses. (Top) Brain images show that (A) the PMBR response and (B) the peak gamma response (MRGS) were both strongest in the left primary motor cortex. Color scale bar in pseudo-t units appears above each image. The time series envelopes (PMBR: 16–22 Hz; MRGS: 72–84 Hz) depict changes in power relative to the baseline at the peak voxels of each response. The yellow box indicates the time window that was significantly different from the baseline period (grey box) based on the MEG sensor-level analyses. (Bottom, A) Results of the mediation model for the PMBR response showed that older age predicted higher testosterone, but there were no further significant assocations. (Bottom, B) Results of the mediation model for the MRGS indicated that older age predicted more testosterone, and higher spontaneous gamma during the baseline predicted weaker MRGS responses. No further mediation effects were significant. Solid lines signify statistically significant relationships at p < .05, dotted lines represent nonsignificant relationships. All reported coefficients are standardized.

The average post-movement beta rebound (PMBR) response was centered on the contralateral primary motor cortex (Fig. 4A). Age was not directly related to PMBR power with the inclusion of other mediating factors in the model, though this relationship was approaching significance (*β* = .30, *p* = .071). There were no other significant associations where developmental metrics predicted baseline or PMBR response power.

### 3.6. Movement-related Gamma Synchronization (MRGS) response

There were two significant effects for the MRGS response in the primary motor cortex (Fig. 4B). Age was positively associated with testosterone (*β* = .72, *p* < .001) and spontaneous gamma power during the baseline was inversely related to MRGS power (*β* = −.47, *p* = .047), such that increased age predicted increased testosterone, and higher spontaneous baseline gamma predicted weaker MRGS response power, respectively. However, we did not detect any indirect effects or relationships between developmental metrics and gamma response variables.

### 3.7. Movement-related Theta ERS responses

**Fig. 5.**
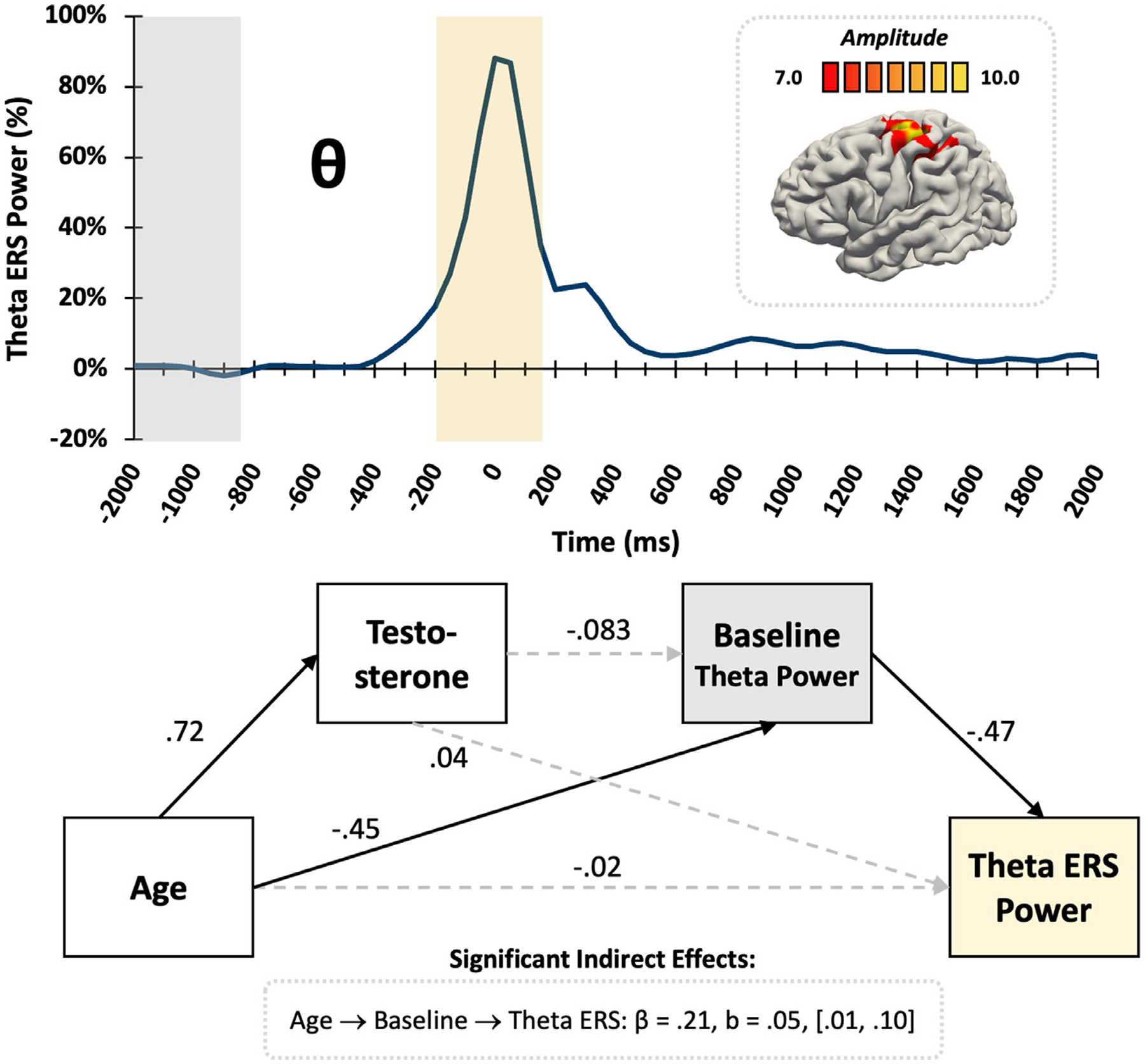
Motor-related theta ERS. (Top) The average brain map shows the theta ERS was strongest in the left motor cortex, with the color scale bar in pseudo-t units directly above. The theta time series envelope (3–6 Hz) extracted from the peak voxel shows a significant increase in theta power (i.e., ERS; yellow box) around movement onset relative to the baseline period (grey box). (*Bottom*) The statistical diagram demonstrates the partial mediation of theta response metrics, with standardized coefficients shown. Solid lines signify statistically significant relationships at *p* < .05, dotted lines represent nonsignificant relationships. The indirect effect of age through spontaneous theta power during the baseline significantly predicted the strength of the theta ERS response.

Statistical analyses revealed that age directly predicted both testosterone (*β* = .72, *p* < .001) and spontaneous theta power during the baseline (*β* = −.45, *p* = .014) in the motor cortex (Fig. 5), with the latter decreasing with increasing age. Subsequently, stronger baseline spontaneous theta power was associated with weaker theta ERS responses (*β* = −.47, *p* = .002). The relationship between age and theta ERS power was fully mediated by spontaneous theta power during the baseline (*β* = .21, b = .05, CI [.01, .10]), as the direct relationship between age and theta ERS power was not significant (*β* = −.02, *p* = .93). Thus, older youth had weaker baseline theta activity and subsequently stronger theta ERS responses.

## 4. Discussion

In this study, we examined the developmental trajectory of neural oscillatory activity serving basic motor control in relation to pubertal testosterone levels in a sample of typically-developing youth. We observed four well known motor-related responses, including the peri-movement beta ERD, the PMBR, and the MRGS and theta ERS during movement execution. All responses localized to the contralateral primary motor cortex, corroborating prior MEG studies of motor-related neural oscillations in youths and adults (Cheyne, 2013; Gaetz et al., 2010; Heinrichs-Graham et al., 2020, 2018a, 2018b, 2017, 2016; Heinrichs-Graham and Wilson, 2016, 2015; Kurz et al., 2016; Spooner et al., 2021b; Trevarrow et al., 2019; Wiesman et al., 2021, 2020; Wilson et al., 2014, 2010). Importantly, we found novel developmental shifts in these oscillatory responses in terms of chronological age and pubertal-specific effects, as indexed by testosterone levels. Mediation analyses revealed complex maturation patterns for the beta ERD response, in which testosterone predicted both spontaneous beta levels and the beta ERD response through direct and indirect effects. Age, but not pubertal hormones, predicted motor-related theta ERS activity, and no relationships between oscillatory outcomes and developmental metrics were found for the PMBR and MRGS responses. Below, we discuss the implications of these findings for understanding the developmental trajectory of cortical motor oscillations during the pubertal transition.

Our most interesting findings were the complex mediation effects of the developmental metrics on peri-movement beta activity. Consistent with previous studies (Heinrichs-Graham et al., 2018b; Heinrichs-Graham and Wilson, 2016; Wilson et al., 2014), participants with the strongest spontaneous beta activity during the baseline also had the strongest beta ERD (i.e., most negative) prior to and during movement, with both direct and indirect effects. Importantly, the mediation analyses indicated the influence of endogenous testosterone levels on these beta oscillations, as higher testosterone was predictive of weaker spontaneous beta activity during the baseline. These findings corroborate prior work showing spontaneous beta power decreases as youth enter later adolescence and early adulthood, potentially indicative of “optimized” resting motor cortices and enhanced neural efficiency (Heinrichs-Graham et al., 2018b). Thus, our mediation analyses contextualize prior literature focused on the effects of chronological age on motor-related oscillations, and show that the effect of testosterone explains the effect of age on beta oscillatory activity, at least to some degree. Beta oscillatory activity is largely dependent on GABA-mediated local inhibitory networks, especially within the primary motor cortices (Hall et al., 2011; Yamawaki et al., 2008). This may suggest a key role of pubertal sex-steroids in the development of these GABA-mediated beta oscillatory responses. The maturation of local GABAergic circuitry continues into adolescence and adulthood (Hashimoto et al., 2009; Kilb, 2012), and studies have shown that sex steroids can influence GABA subunit expression and modulate GABAergic activity (Belelli et al., 2006; Belelli and Lambert, 2005; Fries et al., 2007; Zhang et al., 1999). Previous studies that pharmacologically manipulated the GABA system in human adults observed subsequent changes in baseline spontaneous beta power and beta ERD responses (Muthukumaraswamy et al., 2013). We found that testosterone mediated both spontaneous beta and subsequent beta ERD responses during movement, which supports prior findings of both spontaneous and active beta oscillatory responses being influenced by GABAergic drive (Hall et al., 2011; Muthukumaraswamy et al., 2013). Therefore, the pubertal rise in androgenic hormones and other sex steroids likely contributes to the development of local GABAergic networks in the motor cortices that facilitate changes in the beta oscillatory activity serving motor control.

Interestingly, our findings did not suggest any significant mediation effects of age or testosterone on the PMBR or MRGS responses. Thus, testosterone did not appear to modulate the development of these responses. Prior work has shown that the PMBR and MRGS responses in the primary motor cortices are more GABA-independent (Hall et al., 2011), which may suggest that they would be less affected by the impact of changing pubertal hormone levels on the development of GABA-inhibitory circuits. Of note, our data indicated a trending direct effect of age predicting PMBR power, which would have be consistent with previous studies that used different motor tasks in smaller developmental samples (Gaetz et al., 2010; Trevarrow et al., 2019; Wilson et al., 2010). Since oscillatory motor activity is largely modulated by movement characteristics such as the certainty and complexity of movement (Gaetz et al., 2013; Grent-’t-Jong et al., 2013; Heinrichs-Graham et al., 2018a, 2016; Heinrichs-Graham and Wilson, 2015; Kaiser et al., 2001; Tan et al., 2016; Tzagarakis et al., 2015, 2010; Wiesman et al., 2021, 2020), future studies using more complex and/or cognitively-involved motor tasks may provide clarity into whether the PMBR and MRGS also show pubertal maturational effects under more demanding movement conditions, as the current study was well powered but also involved a relatively simple motor task.

In regard to motor-related theta activity, our mediation analysis showed that chronological age predicted spontaneous theta during the baseline, which subsequently predicted theta ERS, observed through significant direct and indirect effects. Of note, testosterone was not a significant predictor of oscillatory theta power within our mediation models, thus the development of motor-related theta activity likely occurs through a different mechanism distinct from the influence of testosterone. Future studies should further investigate other potential developmental markers, such as DHEA, to determine the main contributors to the development of motor-related theta oscillations.

Before closing, it is important to acknowledge some limitations of the present study. Circulating hormones, such as testosterone, are cyclic in nature and inherently fluctuate between individuals on the bases of sex, time of day, and other situational factors (Herting and Sowell, 2017; Matchock et al., 2007). In the present study, salivary samples were collected at similar times and sample concentrations did not differ by sex, thus these fluctuations potentially had minor effects on our data that were largely accounted for. Nonetheless, future work using alternative hormonal assay methods, such as hair hormone concentrations, could provide a more representative, average level of physiologically active pubertal hormones (Short et al., 2016). Future studies should also investigate the influence of other sex steroids, such as DHEA, progesterone, and estradiol, which dramatically change throughout pubertal development and have the potential to influence other components involved in the maturation of neurophysiological responses and cortical systems. Though the present study utilized a substantial sample size (n = 74), our data was potentially underpowered to detect more subtle patterns of oscillatory development. Specifically, the full mediation of the beta ERD response (age → testosterone → baseline beta power → beta ERD) was trending towards significance, but did not surpass the threshold. Replicating this study in a larger sample would likely confirm these findings and detect other more subtle effects. Lastly, future studies should examine the effect of handedness on motor-related oscillations in developmental samples. We collapsed across this variable due to the limited number of left-handed participants, but differences in the neural dynamics serving left- and right-handed movements have been demonstrated, and developmental studies are warranted (Wiesman et al., 2021).

In conclusion, the current study was the first to investigate the influence of pubertal testosterone levels on the development of cortical oscillations serving motor control in typically-developing children and adolescents. Complex maturation patterns for the beta ERD response were observed, which reflect the major impact of testosterone changes on the development of cortical motor function during the pubertal period. Age-related changes in the theta motor response were also identified, and no significant developmental effects were observed for the PMBR and gamma ERS responses. Overall, these findings highlight the continued maturation of neural oscillations serving motor control and provide novel insight into the influence of pubertal hormones on functional cortical development during the pubertal transition.

## Data Availability

All data that support the findings of this study are available upon reasonable request to the corresponding author (TWW). Data will be made publicly available upon study completion.
